# Limitations of concurrently representing objects within view and in visual working memory

**DOI:** 10.1038/s41598-020-62164-y

**Published:** 2020-03-24

**Authors:** Tengfei Liang, Zijian Cheng, Wenjing Hu, Chaoxiong Ye, Jiafeng Zhang, Qiang Liu

**Affiliations:** 1 Institute of Brain and Psychological Sciences, Sichuan Normal University, Chengdu, 610000 China; 2 Research Center of Brain and Cognitive Neuroscience, Liaoning Normal University, Dalian, 116029 China; 3 Department of Psychology, University of Jyvaskyla, Jyväskylä, 40014 Finland; 4 CAS Key Laboratory of Behavioral Science, Institute of Psychology, Chinese Academy of Sciences, Beijing, 100101 China

**Keywords:** Perception, Perception, Working memory, Working memory, Human behaviour

## Abstract

Representing visibly present stimuli is as limited in capacity as representing invisible stimuli in visual working memory (WM). In this study, we explored whether concurrently representing stimuli within view affects representing objects in visual WM, and if so, whether this effect is modulated by the storage states (active and silent state) of memory contents? In experiment 1, participants were asked to perform the change-detect task in a simultaneous-representing condition in which WM content and the continuously-visible stimuli in view were simultaneously represented, as well as a baseline condition in which only the representations of visual WM content were maintained. The results showed that the representations in visual WM would be impaired when the continuously-visible stimuli in view were concurrently represented, revealed by the reduced CDA amplitude and the lower behavior performance. In experiment 2, a dual-serial retro-cue paradigm was adopted to guide participants to maintain memory items in two different storage states, and the results revealed that simultaneously representing the continuously-visible stimuli and the WM content would only impair the WM representations in the active state. These evidences demonstrated that only the visual WM representations that were maintained in the active state would definitely share the limited resources with the representations of continuously-visible information, and further supported the dissociation between the active state and silent state of visual WM storage.

## Introduction

Visual working memory (WM) supports the reconstruction of mental representations of objects in the brain without perceptual input. Limited storage capacity is the most striking feature of visual WM, enabling individuals to represent only a few WM items at once^1,2^. In everyday perception, more often than not, visual inputs we are representing are always in view. These continuously visible representations do not need to be reconstructed in our minds, thus making the short-term storage unnecessary. Intuitively, it seems that the more continuously-visible information can be simultaneously represented compared to that is no longer visible to the observers but was maintained in visual WM. For example, we can easily scan the entire playground, but it seems difficult to recreate the image of whole scene in WM. However, counter-intuitively, there is broad agreement in the literature that people can only represent a small number of objects within view at the same time, showing limited capacity which is commonly observed in visual WM. This was observed in the object recognition^3,4^ and multiple object tracking task^5–7^, in which objects were always presented within view. The fact that representing information within view and in visual WM both showed similar limited capacity, only 3–4 items^1,8–10^, raised an interesting speculation that they may be subject to the same capacity mechanism or belong to the same “representational” system.

Evidences at the neurological level support this speculation. Specifically, the anatomical structure of the visual cortex may limit representing multiple visual stimuli simultaneously, such as the topological relationship with retina, whether they are visibly present or not. On the one hand, neuroimaging studies have firmly established that representing stimuli within view could lead to competitive inhibition between them in the visual cortex, especially when cortical spaces are adjacent^11,12^. On the other hand, according to the influential sensory recruitment account, the visual cortex, which was initially responsible for the encoding of the memory items, also participates in short-term storage of them^13–15^. For example, multiple pieces of evidence from the multivariate analyses of neural representations suggest that representing WM items in visual areas helps maintain them with high fidelity^16,17^. Thus, visual cortex is recruited for representing information both within view and in visual WM. This makes an adverse situation possible, that is, when concurrently representing objects within and without view, the two may compete with each other in the early sensory areas. As a result, the active maintenance of visual WM would be impaired, resulting in the reduced fidelity or the information loss caused by the competition.

A recent study directly explored the relationship between representing objects within view and in visual WM. Tsubomi *et al*.^10^ asked participants to perform a color change detection task with two different conditions: “absent” condition and “present” condition. For the former, the stimuli array was presented for 100 milliseconds (Representing objects in visual WM) and followed by a 900 milliseconds delay interval (i.e., the retention stage of WM) before the test display was presented. For the latter, the stimuli array was presented for 1000 milliseconds (Representing objects within view) without a delay interval, and followed by a test display immediately. They were surprised to find that the same limited capacity was observed in both the “absent” and “present” conditions. That is, the number of objects that could be represented by the participants was consistent, at most 4 items, regardless of whether they were visibly present or not. Moreover, the capacity under the two conditions was significantly correlated at the individual level. These findings led the authors to speculate that there may be a “representational” mechanism with limited capacity involved in representing information, regardless of whether it was within view or not. Here, we further tested a direct prediction of this view in experiment 1, that is, representing stimuli remained continuously in view would compete with representing items in visual WM for limited “representational” resources.

Another issue of concern in current research is that, if representing visibly present stimuli interferes with representing items in visual WM, whether this effect is modulated by the storage states of memory contents? This question is mainly based on the state-based models of WM which considered that WM items could be stored in either active state or the silent state. Those selected memory items are retained in the active state, which has a high degree of accessibility; while the others are temporarily stored in the silent state^18–20^. Several laboratories have provided neurological evidence for distinguishing between two states^21,22^. Interestingly, researchers consistently found that the visual cortex only represented memory contents maintained in the active state^22,23^. While the memoranda of the silent state relied on the brain regions outside the sensory cortex, such as the parietal cortex^24^, frontal area^25^, or through the activity-silent memory mechanisms (e.g., short-term synaptic plasticity)^25–27^. Notably, as mentioned above, representing stimuli continuously presented in view is highly dependent on the involvement of the visual cortex. Therefore, experiment 2 was performed to test the hypothesis that visual WM items stored in the active state, rather than the silent state, were more likely to be affected when the visibly-presented stimuli was represented simultaneously during the WM maintenance period.

## Experiment 1

The aim of experiment 1 was to examine whether simultaneously representing stimuli within view affected representing items in visual WM. To do this, we borrowed the paradigm created by Tsubomi *et al*.^10^ and designed a simultaneous-representing condition in which participants were asked to represent information both within view and in visual WM during the delay interval. We compared the memory performance in this condition with the baseline condition in which participants only need to represent items in visual WM (i.e., traditional change detection task^28,29^). EEG data were also collected during the experiment. In order to obtain pure neural activity associated with visual WM, we presented the stimuli within view on the midline of the screen. Under this design, any lateralized neural activity observed during the delay interval would just reflect the processing associated with visual WM. CDA amplitudes were measured during the delay interval because it can reflect the real-time storage of visual WM^30,31^, and track the fluctuations of memory performance trial-by-trial^32^. If representing stimuli remained continuously in view competed for resources that was originally used to represent memory items in VWM across a delay, then the CDA amplitude and behavior memory accuracy should be significantly lower under the simultaneous-representing condition than the baseline condition, which reflected impaired representational quality of WM items.

Besides, based on the assumption that the same limited resource pool is used for representing objects within view and in visual WM, it can be expected that the two conditions should be allocated equal amounts of “representational” resources. This means that the amount of information that an individual can represent at the same time should be the same under both conditions, regardless of whether the information is visible or not. We further hypothesized that this limited “representational” resource might come from the attentional control of the frontal network. To test this idea, we measured the frontal theta (4–8 Hz) power under different conditions as an oscillating marker of attentional control^33,34^.

## Methods

### Participants

Twenty-four neurologically normal volunteers (eleven males, mean age 22.74 years, 17–28 years old) participated in the experiment 1 for monetary compensation. One participant was rejected due to the excessive noise in the EEG data. Informed consents have been obtained from all the participants. For the participant who was 17 years old, informed consent was obtained from his parent. The study was approved by the Ethics Committee of the Liaoning Normal University, China, and conducted in accordance with the Declaration of Helsinki (2008).

### Apparatus and stimuli

The stimuli were presented with E-Prime 2.0 (Psychology Software Tools, Inc., Pittsburgh, PA) on a LCD screen (60 Hz refresh rate) against a gray background, with a black fixation cross (0.19°) appearing constantly throughout each block. Participants were seated in a semi-dark room at a viewing distance of 90 cm. Participants maintained the fixation on a small black dot (0.12°). Each of the stimuli array consisted of six colored squares randomly drawn from a list of eight colors (blue, magenta, black, lime, red, cyan, white, and yellow). Each square subtended 1.2°. There were six fixed positions of the stimuli display (the left and right visual fields contained two positions each, with the distance of two adjacent squares was 3.6°, and the other two are presented above and below the fixation cross), which were arranged in an imaginary circle with a radius of about 4.95°.

### Procedure

**Figure 1:**
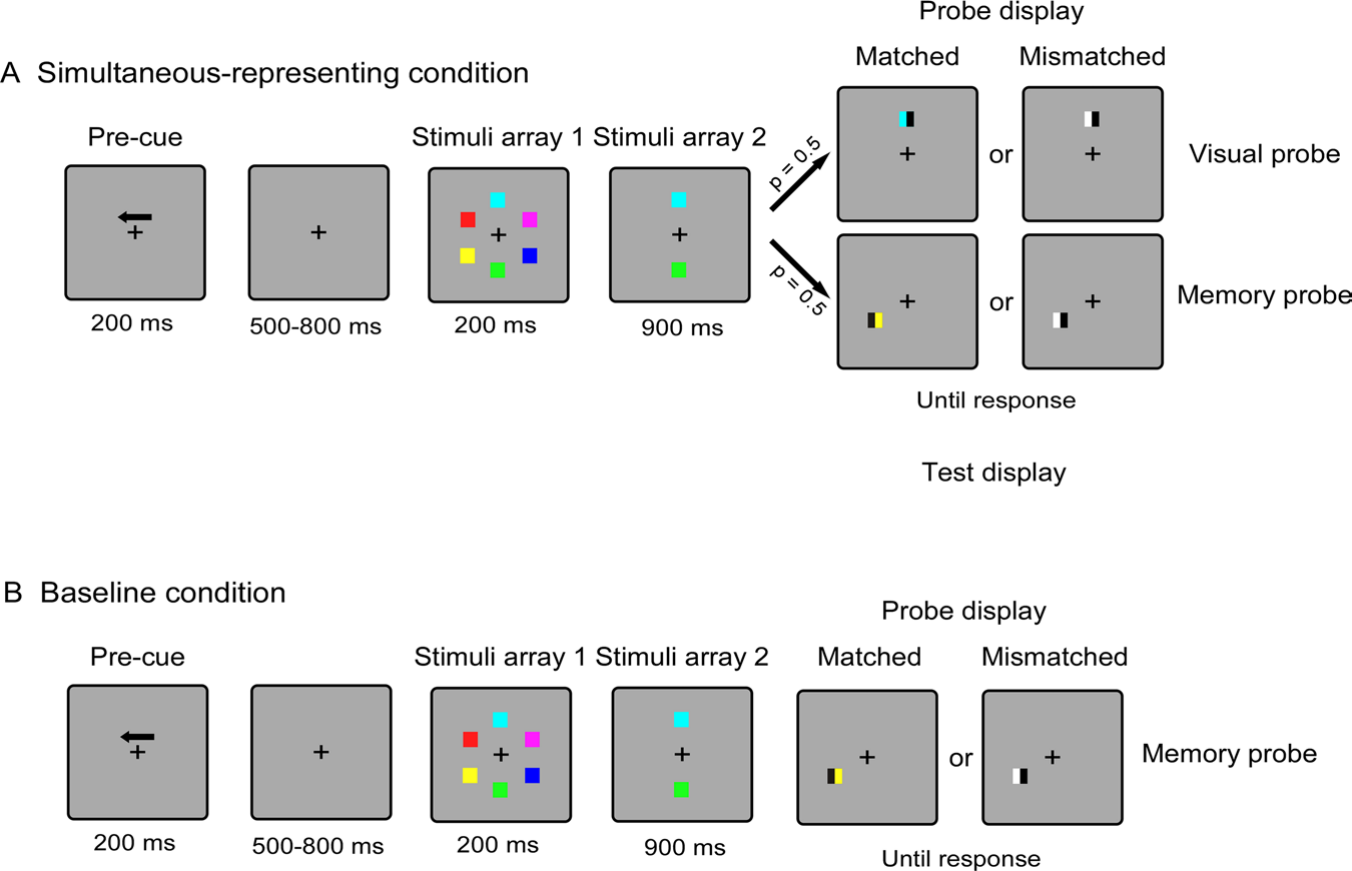
Experiment 1 task. Schematic illustration of the trial for the (**A**) simultaneous-representing condition and (**B**) baseline condition. Noting that the main difference between the two conditions is whether the visual probe was included. Under the simultaneous-representing condition, the probability of occurrence of two kind probes was the same. While under the baseline condition, only the memory probe was presented, and the participants were told in advance to ignore the persistently visible stimuli presented on the central axis.

As depicted in Fig. 1, at the beginning of each trial, a central arrow as the cue (200 msec) instructed the participants to covertly memorize the colors of the squares in either the left or the right side. After a random interval of 500–800 msec, six colored squares were presented for 200 msec (stimuli array 1), followed by a retention interval of 900 msec during which the central-presented stimuli (stimuli array 2) still remained on the screen until the test display was presented, but the laterally-presented memory items disappeared. Then, the test display was presented until making a response. The current experiment contained two different sessions corresponding to two conditions: the simultaneous-representing condition and the baseline condition, and the session order was counterbalanced across participants. For the simultaneous-representing condition (see Fig. 1A), When the probe display was presented, a memory probe was presented at one of the previous locations of the laterally-presented memory items, while the visual probe was presented at one of the locations of the centrally-presented stimuli. Since the lateralized memory items were presented for only 200 msec, participants must maintain these items in mind to response accurately at the detection stage. At the same time, they also had to keep staring at the centrally-presented stimuli during the delay phase, in response to the visual probe that might be presented on the test display, because the memory probe and the visual probe were randomly presented with the 50% chance each. For the baseline condition (see Fig. 1B) in which only the memory probe would be presented, participants just needed to memorize the lateralized items, which was designed as a pure visual WM task, and the Centrally-presented stimuli were required to be ignored because they were task-irrelevant. The probe item in the two conditions was consisted of two rectangles with half the width of the color square. In the trials of “unchanged” probe, one of the rectangles has the same color as the square at that position, and the other one was filled with a new color. In the trials of “changed” probe, both rectangles were filled with new colors. When the probe was presented, participants needed to detect whether one of the two rectangles has the same color as the memory stimuli at that position. The “changed” and “unchanged” trials corresponded to different keys respectively (“M” and “Z” key), and the two kinds of trials were presented randomly with equal probability (50%). Participants gave an unspeeded response. The next trial began following a 1000 ms-interval delay after the participants made response.

Each session was provided with an appropriate instruction to ensure that the participants knew whether the centrally-presented stimuli were task-relevant in advance. There was a break of at least 1 min between blocks and 4 min between sessions, with one practice block (at least 12 trials) before each session. All participants completed a total of twelve blocks of 48 trials each, resulting in 288 trials per condition.

### EEG data acquisition and analysis

EEG data were acquired using an ANT-NEURO system (Enschede, The Netherlands) with 64 Ag/AgCl electrodes arranged in a 10/20 system layout (including left and right mastoids, AFz serving as ground, and CPz serving as the on-line reference) at a sampling frequency of 500 Hz. Horizontal eye movements were recorded by electrodes placed on the outer canthi of the right and left eyes, and vertical eye movements were recorded from Fpz electrode to detect blinks.

Electrophysiological signals were firstly filtered with 40 Hz low-pass and re-referenced to an average of the mastoids, and then epoched from −500 msec to 1500 msec around the onset of stimuli array 1. Trials with incorrect or missing responses were excluded from further analyses. Split-half sliding window approach was used on the VEOG electrodes to identify saccades (window size = 200 msec, step size = 10 msec, threshold = 70 μV) and on the HEOG electrodes to reject gaze drift (window size = 200 msec, step size = 10 msec, threshold = 20 μV). In addition, segments were excluded from further analysis when the absolute voltage of the interested electrodes (F3, F4, Fz, PO5/PO6, and PO7/PO8) exceeded 80 μV. Participants were excluded from further analyses if, after all of the artifact rejection procedures, the remaining number of EEG trials per condition was less than 75 trials. Based on this criterion, one participant was rejected.

For the CDA analysis, EEG trials (processed data) were firstly baselined over the 200 msec before the memory array. Then, ERPs were obtained by averaging EEG trials of two adjacent posterior electrodes (PO5/PO6 and PO7/PO8), separated for the hemispheric contralateral and ipsilateral to the memory arrays on the task-relevant side. Finally, CDAs, a kind of difference waveforms, were calculated by subtracting the ERPs of the ipsilateral electrodes from the ERPs of the contralateral electrode.

For the frontal theta power analysis, the single-trial EEG signal (processed data) was convolved with complex Morlet’s wavelets^35^. Using the “cwt.m” function in the wavelet toolbox in MATLAB, instantaneous power was extracted from the entire epoch. We computed instantaneous power by taking square of the complex magnitude of the complex analytic signal. Percent change of the instantaneous oscillatory power values was firstly calculated relative to the baseline period data (−400 to −100 msec relative to stimuli array 1 onset). Then, frontal theta activity was acquired as the average of theta band power (4–8 Hz) in the frontal electrodes (F3, F4, and Fz)^32^.

To determine subtle statistical significance and investigate the temporal dynamics of the effects of representing visibly present stimuli on representing visibly absent memory items, the time series for CDAs and frontal theta power waveforms for both the simultaneous-representing condition and the baseline condition were tested against chance and also against each other at the group level using non-parametrical cluster-based permutation test^36^.

The behavior data were analyzed by independent sample and paired sample t test. *Cohen’s d*^37^ and its confidence interval^38^ were reported to provide statistical power.

## Results and Discussion

### Behavioral results

**Figure 2:**
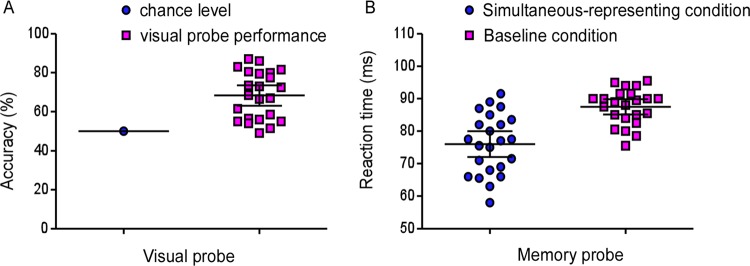
Dots represent the behavioral results of the single participant in Experiment 1 task. (**A**) Percentage correct of the visual probe trials, compared with the chance level. (**B**) Percentage correct of the memory probe trials for both the simultaneous-representing and baseline conditions. The horizontal line in the middle represents the group mean. Error bars represent the 95% confidence interval.

We firstly tested whether participants could actively represent the visibly-presented stimuli when they were maintaining visibly-absent items in visual WM in the simultaneous-representing condition. A single sample t test was adopted to analyze. As shown in Fig. 2A, the results showed that the percentage correct for visual probe was 68.39 ± 2.54%, which was significantly higher than the chance level, *t* (22) = 7.24, *p* < 0.001, *cohen’s d* = 1.51, 95% CI = [0.898, 2.1], demonstrating that participants were engaged in the task.

In the current experiment, it could be assumed that the continuously-visible stimuli in view would compete with the representations of visual WM content for the limited “representational” resources, which lead to a clear prediction that the performance of the memory probe would be significantly impaired in the simultaneous-representing condition compared to the baseline condition. A paired sample t test was used to test this hypothesis. As shown in Fig. 2B, the results showed that the memory accuracy in memory probe trials was significantly low under simultaneous-representing condition (76.0 ± 1.95%) compared to baseline condition (87.48 ± 1.13%), *t* (22) = −6.23, *p* < 0.001, *Cohen’s d* = −1.30, 95% CI = [−1.85, −0.73] (see Fig. 2B), indicating that representing the continuously-visible stimuli in view would affect the visual WM performance.

## Electrophysiological Results

### CDA

**Figure 3:**
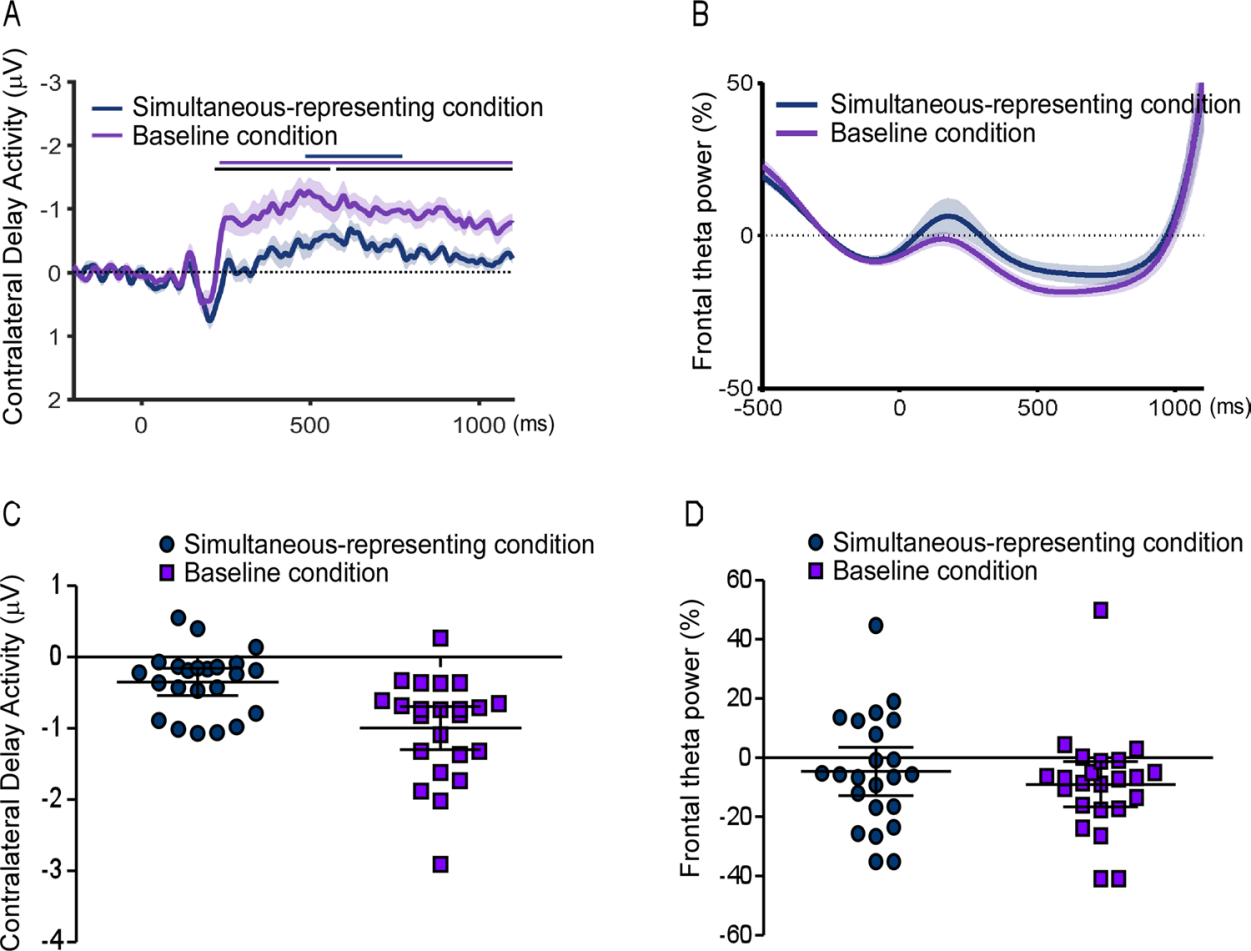
On the top, time series of the contralateral delay activity (**A**) and frontal theta power (**B**) for both the simultaneous-representing and baseline conditions relative to the baseline interval (−400 to −100 ms before the onset of the stimuli display) on the Experiment 1 task. Time point zero indicates the stimuli display onset. Lines with different colors along the top of CDA plot indicate the data points at which either the difference of both neural indicators between conditions (black) or the neural indicators itself for each condition (dark blue for simultaneous-representing condition and purple for baseline condition) were significantly different than zero (p < 0.05, cluster corrected). Shaded error bars indicate 1 SEM. On the bottom, dots represent single participant’s data of the contralateral delay activity (mean amplitude in the 300–1100 ms interval after the onset of the stimuli display) (**A**) and the frontal theta power (mean amplitude in the 300–1100 ms interval after the onset of the stimuli display) (**B**). The horizontal line in the middle represents the group mean. Error bars represent the 95% confidence interval.

The behavioral data above indicated that simultaneously representing visible items compromised the performance of the visual WM. However, the evidence at the behavioral level could not rule out the possibility that reduced memory performance might result from the decision interference during the detection phase under the simultaneous-representing condition. Notably, our hypothesis considered that the effect of representing visible items on visual WM information occurred during the maintenance phase. Hence, it remained unclear whether the WM performance was impaired during the retention stage at the neurological level. CDA components was analyzed, which were widely used as the neural marker of online storage^31,32^. The cluster-based permutation test was used to reveal the statistical difference of CDA amplitude between two conditions. As shown in Fig. 3A, the results of analysis revealed that a significant CDA was observed during the retention stage for both the simultaneous-representing condition (significant time points: 12 to 128 msec and 150 to 242 msec, cluster-defining threshold p < 0.05, corrected significance level p < 0.05) and baseline condition (significant time points: −78 to 148 ms and 314 to 804 msec, cluster-defining threshold p < 0.05, corrected significance level p < 0.05). Importantly, the CDA amplitude under the simultaneous-representing condition was significantly lower than that under the baseline condition (significant time points: 220 to 1100 msec, cluster-defining threshold p < 0.05, and corrected significance level p < 0.05). This finding further complemented the conclusion from the behavioral data and ruled out an alternative explanation, which confirmed that participants might not always represent the continuously-visible stimuli, but rather store them in mind before the onset of the test display. Therefore it could be expected that CDA amplitude would not decrease, or if any, the decrease of CDA appeared just before the probe array onset.

### Frontal theta power

The frontal midline theta activity was measured to explore whether more cognitive control was involved in simultaneous-representing condition than baseline condition. As shown in Fig. 3B, it could be seen that the difference of frontal theta power between the two conditions was not significant, proved by the non-parametrical statistical analysis. In the time window of the whole period, the difference of frontal theta power between conditions did not reach the significance. (cluster-defining threshold p < 0.05, corrected significance level p < 0.05).

It should be pointed out that the two conditions were performed in separate blocks and participants were told whether the visual probe would be detected in advance. Therefore, participants did not need to use memory strategies, such as storing the continuously-visible stimuli in mind to complete the detection of visual probes in the baseline condition. However, the memory strategy was optimal for the memory probe task in which the sample array was expected to disappear after a few hundred milliseconds, and followed by a retention interval^28,29^. Indeed, some visual tasks have been thought to primarily involve visual-spatial processing and also recruit the visual WM system. For example, the previous location of searched item need to be maintained in visual search task, which prevented the re-searching for them^39,40^. In contrast, representing continuously-visible stimuli in current experiment would not potentially require participants to store then into the WM, but only needed to maintain static information about the central-presented stimuli within view with no additional processes.

## Experiment 2

The results of Experiment 1 showed that simultaneously representing objects within view impaired representing items in visual WM. In experiment 2, we further tested whether this effect only applies to memory items stored in the active state, rather than the items in the silent state. To do this, a dual-serial retrocue paradigm was conducted to manipulate the storage state of memory items. In this paradigm, the cued items would be maintained in the active state during the delay interval after the first retro-cue. While the uncued items were maintained in the silent state because they were currently task-irrelevant but probed in the second memory probe. Behavioral and neurological evidence from multiple researches have confirmed the validity of this paradigm^21,22,27,41^.

### Participants

A new set of thirty-six participants (twenty-one females, mean age 20.72 years, 19–25 years old) with normal color vision and visual acuity completed the experiment 2 for monetary compensation. All participants were provided with informed consent before experiment, and all procedures were approved by the Ethics Committee of the Liaoning Normal University, China, and conducted in accordance with the Declaration of Helsinki (2008).

### Stimuli and procedure

**Figure 4:**
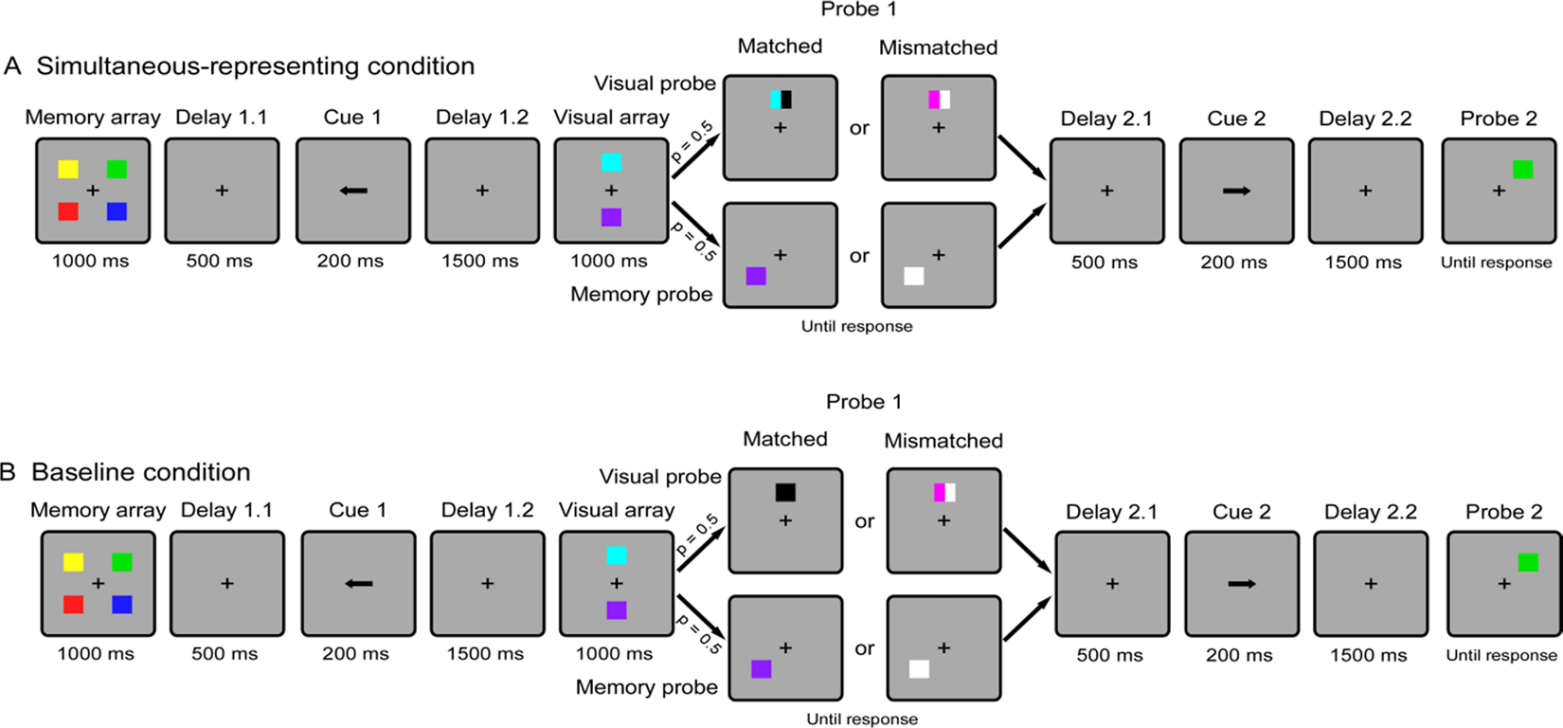
Experiment 2 task. Schematic illustration of the trial for the (**A**) simultaneous-representing condition and (**B**) baseline condition. Noting that the difference between the two conditions is mainly the display of the visual probes and corresponding task requirements.

Experiment 2 consisted of two different sessions, each corresponding to a condition, and the session order was counterbalanced across participants. On each trial of the simultaneous-representing condition (see Fig. 4A), participants were first presented with a memory array for 1000 msec, consisting of four colored squares (parameters identical to those in the experiment 1) located left and right around a central fixation cross. Subsequently, the first retro-cue was presented for 200 msec following a delay interval of 500 msec (Delay 1.1) after the offset of the memory array. The retro-cue (an arrow) was presented in the center of the screen, pointing to either the left or right side to indicate which two items would be probed in the first test display. After a delay interval of 1500 msec (Delay 1.2), two new colored squares appeared for 1000 msec above and below the fixation point (visual array), followed immediately by a test display (Probe 1). There were two different scenarios in the test display, each with a probability of 50%. In memory probe trials, participants were presented with a probe in one of the locations (randomly selected) previously occupied by the cued memory items. The probe remained on screen until participants decided whether it matched the color of the memory item that had been presented in the same position by pressing the “Z” key for a match response or the “M” key for a mismatch response. In visual probe trials, a probe item, which consisted of two rectangles with half the width of the squares, was presented at one of the locations of the centrally-presented colored squares. In the trials of “matched”, one rectangle remained the same color as the previous squares presented in the same location, and the other one was filled with a new color. In the trials of “mismatched”, both rectangles were filled with new colors. This probe item remained on screen until participants decided whether one of the two rectangles had the same color as the previous stimulus presented in the same location by pressing the “Z” key for a match response or the “M” key for a mismatch response. Probe 1 was followed by a third delay period (Delay2.1) of 500 msec. After that a second retro-cue was presented for 200 msec, indicating that the other two items would be probed in probe 2. After an interval of 1500 msec (Delay 2.2), the second test display (Probe 2) was presented, similar to the memory probe of probe 1.

In the baseline condition (see Fig. 4B), the procedure was similar to the that used in the simultaneous-representing condition, with the exception that participants were faced with a completely different task requirement in the first test display of the visual probe trials. When the visual probe was presented, it remained on screen until the participants decided whether it was composed of two rectangles of different colors by pressing the “M” key for “yes” or pressing the “Z” key for “no”. Therefore the centrally-presented stimuli were totally task-irrelevant under this condition, and they would be ignored. The proportion of the visual probe trials and the response pattern in the baseline condition were the same as the simultaneous-representing condition. The probability of match response and mismatch response in each type trials was randomly presented with 50% chance.

To discourage verbal encoding of the memory array, a randomly selected four-digit number was presented at the beginning of each block. Participants were required to rehearse them subvocally throughout each trial.

Each condition was provided with an appropriate instruction to ensure that the participants knew whether the centrally-presented stimuli were task-irrelevant in advance. There was a break of at least 1 min between blocks and 4 min between sessions, with one practice block (at least 12 trials) before each session. All participants completed a total of eight blocks of 30 trials each, resulting in 240 trials in total.

## Results and Discussion

### Memory accuracy

**Figure 5:**
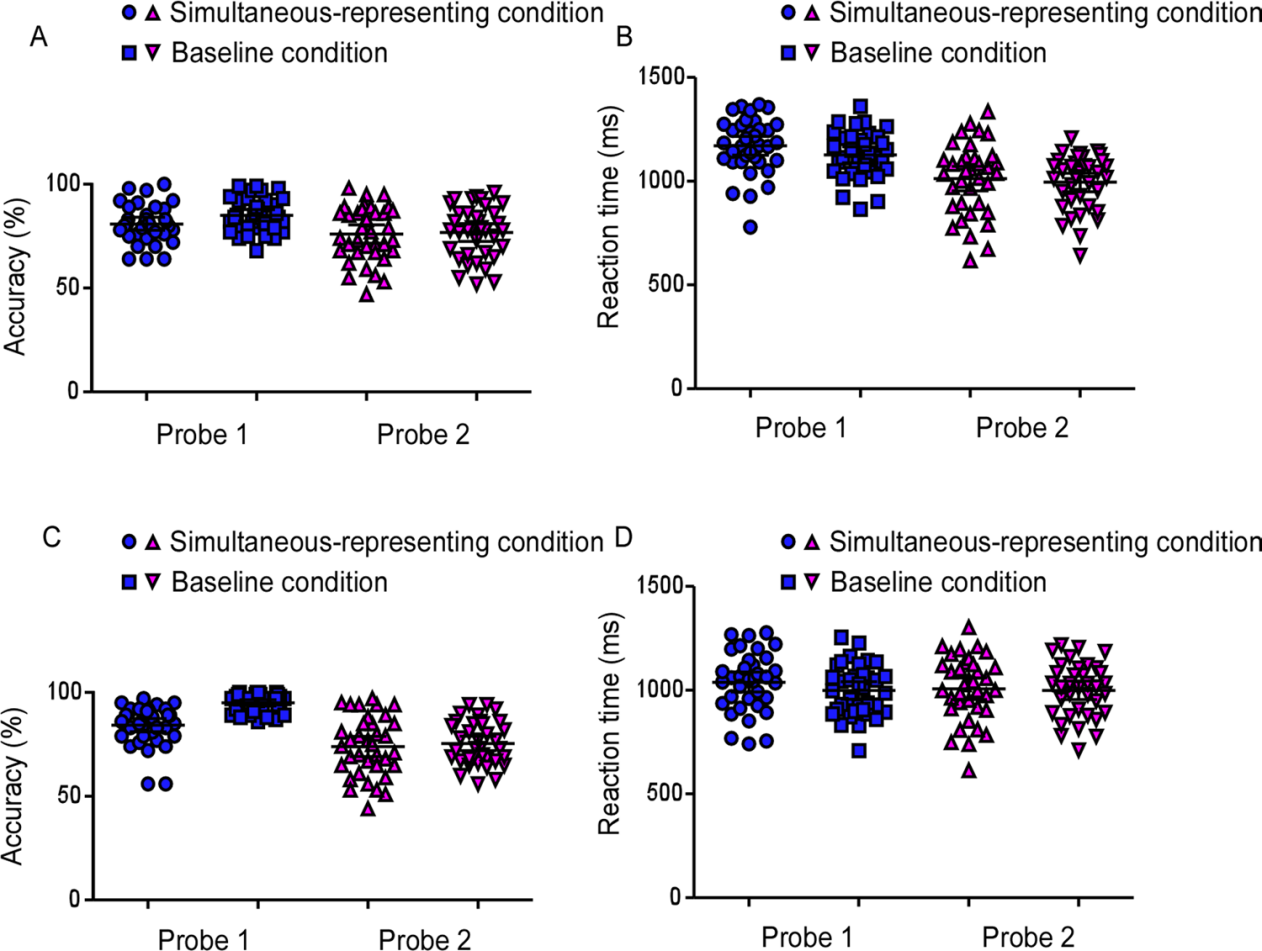
Dots represent single participant’s behavioral results of the Experiment 2 task. On the top, Percentage correct (**A**) and Reaction times (**B**) are plotted when probe 1 was the memory probe trials for both the simultaneous-representing and baseline conditions. On the bottom, Percentage correct (**C**) and Reaction times **(D**) are plotted when probe 1 was the visual probe trials for both the simultaneous-representing and baseline conditions. The horizontal line in the middle represent the group mean. Error bars represent the 95% confidence interval.

Figure 5A presents the memory accuracy in each condition of Experiment 2. In the current design, probe 1 could detect the active state of WM representations and probe 2 could provide the detection of the silent state of visual WM content. We were specifically interested in examining whether representing stimuli within view during the delay interval would differently affect the WM representations in different states. To this end, we performed a repeated measures ANOVAs with the factors condition (simultaneous-representing condition *vs*. baseline condition) and probe order (probe 1 *vs*. probe 2). It should be pointed out that only the trials in which probe 1 was the memory probe were analyzed, because the cognitive processing involved in these trials was totally consistent in the two conditions, except the requirement of representing the continuously-visible stimuli. This analysis revealed a significant main effect of condition, *F* (1, 35) = 7.01, *p* < 0.05, *η*^2^ = 0.17, probe order, *F* (1, 35) = 31.08, *p* < 0.001, *η*^2^ = 0.47, and a significant interaction, *F* (1, 35) = 4.79, *p* < 0.05, *η*^2^ = 0.12. Planned comparisons revealed a significant difference between simultaneous-representing condition and baseline conditions for probe 1, M-dif = −4.1%, SE-dif = 0.8%, *t* (35) = −5.13, *p* < 0.001, *Cohen’s d* = −0.86, 95% CI = [−1.23, −0.46], but not for probe 2 (t < 1). These results indicated that only the visual WM representations in the active state would be affected if WM content, accompanied with the visible-presented stimuli were simultaneously represented, but not those in the silent state.

Presumably, there should no significant difference of the accuracy of WM representations in the silent state (i.e. the probe 2) between two conditions, even when the first probe was visual probe. We performed a planned contrast to verify this conjecture, analyzing those trials in which the probe 1 was the visual probe and examined the difference of accuracy of probe 2 between the two conditions. Consistent with our hypothesis, the difference failed to achieve the significance, *t* (35) = 1.132, *p* = 0.27. In addition, a paired-samples *t* test was performed to examine whether there was any difference in the performance of visual probe under two conditions, and the results showed that the accuracy of the visual probe in the simultaneous-representing condition was significantly worse than that of the baseline condition, M-dif = 10.78%, SE-dif = 1.7%, *t* (23) = 6.26, *p* < 0.001, *Cohen’s d* = 1.04, 95% CI = [1.45, 0.63], (see Fig. 5C). This result was predictable because the centrally-presented stimuli that was visibly-presented in view were necessarily represented and then competed for the capacity, and the previous study has confirmed that this processing is limited in capacity^10^.

In addition, the accuracy of probe 2 was analyzed in each condition and made a comparison with the chance level (50%) respectively. The results showed that the performance of probe 2 under both the simultaneous-representing condition and the baseline condition was significantly higher than the chance level, regardless of whether the probe 1 was a visual probe or a memory probe, indicating that the memory items stored in the silent state were successively maintained in mind (all *p* < 0.001, *Cohen’s d* ≥ 1.681).

### Reaction times

Reaction Times (RTs) of incorrect responses, faster than 200 msec and slower than 2,000 msec, as well as those exceeding a participant’s mean by more than three standard deviations for each design cell were excluded from the RT analyses. Figure 5B showed the results of RTs. The RTs data were processed with the same analyses as the memory accuracy data. A 2×2 repeated measures ANOVA revealed a significant effect of probe order, *F* (1, 35) = 100, *p* < 0.001, *η*^2^ = 0.74, and a marginal significance of condition, *F* (1, 35) = 3.8, *p* = 0.058, *η*^2^ = 0.10. However, the interaction between the probe order and the condition was not significant, *F* (1, 35) = 2.27, *p* = 0.14, *η*^2^ = 0.06. Therefore, the effect of representing visibly-presented stimuli on VWM representations was not manifested on the response speed. This finding also indicated that the differences in memory performance could not be explained by a trade-off between speed and accuracy. Consistent with the analysis of memory accuracy, two other planned contrasts was performed and showed no significant difference (all *p* > 0.163) (see Fig. 5D).

The evidences provided by experiment 2 rule out three straightforward alternative explanations for the findings in experiment 1. First, participants were given sufficient consolidation time (in this experiment, the memory array was presented in 1000 msec) compared to experiment 1. According to Woodman and Vogel’s data, it took about 50 msec to consolidate a colored square^42^. Thus, participants were able to consolidate all items into visual WM before the first retro-cue was presented in this experiment. Second, participants were required to concurrently articulate a four-digit number to prevent them from verbally recording and rehearsing the memory items^43^. Third, compared to experiment 1, the probability that the first probe was a memory or visual probe was equal in this experiment, i.e. 50%. Thus, the results of the first memory probe at this experiment could not be explained by the detection noise generated by the expectation of visual probe. Therefore, the dissociation between the active and silent states of visual WM storage did not result from the inadequate consolidation, verbally rehearsing, or the decision noise, without appealing to the idea of a limited “representational” resources being shared between representing information remained continuously in view and those retained in the active state of visual WM.

## General Discussion

Growing evidence in the literature showed that the amount of visual information people could simultaneously represent, whether within view or in WM, was limited. The current study examined two hypotheses related to this phenomenon. The first was whether representing visibly present information may compete for resources to represent items in visual WM. Second, considering the state-based model of WM, we tested whether this competition had a distinct effect on memory items stored in the active state and silent state. To test the first hypothesis, in experiment 1, we created a simultaneous-representing condition in which simultaneously representing items that were either remained continuously within view or disappeared across a delay interval (i.e. the WM items) would compete with each other. The results showed that memory performance in simultaneous-representing condition was impaired compared to the baseline condition in which only the visual WM task was necessarily performed. Additionally, EEG data revealed that the CDA amplitude in the simultaneous-representing condition was lower than that in the baseline condition, suggesting that real-time storage of memoranda had been impaired during the retention stage. Those findings are well comparable to that of Tsubomi *et al*.^10^. They found that representing both visibly present stimuli and visibly absent memory items showed similar capacity limits. Our data further confirmed that both of them may use the same resource pool with limited capacity.

In experiment 2, we explored the second hypothesis of this study by using a dual-serial retrocuing paradigm to manipulate the storage state of WM state. The results showed that WM performance was still impaired when the inadequate consolidation, verbally recording and decision noise were excluded, consistent with the findings of experiment 1. Importantly, the data from experiment 2 also showed that competition from the simultaneous representations of continuously-visible items and the invisible memory items only affected the memory performance of items that retained in the active state, not the silent state. This finding indicated that the impaired memory performance observed under the simultaneous-representing condition was unlikely to be due to the general dual-task effect, because the impairment of WM performance was only derived from the items retained in the active state.

Tsubomi *et al*. pointed out that a “representational” mechanism with limited capacity is engage in representing information, whether it is visibly present or invisible in visual WM^10^. However, the intuitive impression given by their account is that visual WM does not seem to be an independent system, but a limited capacity “representational” mechanism for the operation of information that are no longer visible. Based on the state-based model of WM, the current evidence here may help explain this dilemma. In experiment 2, we found that only the active state of visual WM might be dominated by the “representational” mechanism, which helps individual to recreate the image of the memoranda in mind in order to cope with the task at hand, while the silent state was not. This is consistent with the idea that the temporal retention and active manipulation of WM contents can operate independently^44,45^. When it is necessary to represent the visible information in the external environment, the information in WM could be transferred to the silent state for temporary maintenance. Researchers have confirmed that memory representations can switch flexibly between the active and silent states. Lewis-peacock and her co-authors found that the neural activity pattern of the WM representations in visual cortex dropped rapidly to baseline levels when they were irrelevant to the current task^21,22^. Crucially, however, the neural signal of the WM representation could be reactivated when they were task-relevant again. In other words, they actually observed a series of transitions of the memory representations from the active state to the silent state and then to the active state again. This mechanism enables the short-term maintenance of memoranda to run effectively when the “representational” mechanism is occupied.

Next we would like to discuss about the neural substrates that underlay the “representational” mechanism. In other words, what resources did representing information both within view and in the active state of visual WM competed for? According to Tsubomi *et al*.^10^, the “representational” resources available under both conditions should be equal, which makes the information that individuals can represent simultaneously in the two conditions consistent, regardless of whether the information is visible or not. We hypothesized that this limited “representational” resource might be derived from the attention control that was dominated by the frontal network. This takes into account the fact that top-down attention control played an important role in perception and visual WM processing^46–48^. If so, we infer that both conditions should consume the same amount of attentional control resources, regardless of whether the representations were consisted of a mixture of visible and no-longer visible objects in the simultaneously-representing condition, or all of the representations were consisted of no-longer visible objects alone in the baseline condition. Considering the previous studies, the frontal theta power was measured under the both conditions as an oscillating marker of the attentional control. The frontal theta was analyzed by collapsing data from both the contralateral and ipsilateral trials, reflecting the processing of all information presented in both sides of the visual field. As expected, we observed the same amount of frontal theta power under two conditions. Besides, we also need to consider another popular explanation that frontal theta activity may reflect the need for conflicting control^33,34^, which could be manifested by the requirement of processing the multiple tasks simultaneously in the current study. For example, participants were encouraged to concurrently represent objects not only within view but also in visual WM under the simultaneously-representing condition. If so, the result of frontal theta power would seem to imply that performing the task would not recruit more conflicting control in the simultaneously-representing condition than the baseline condition.

Another possibility discussed here was that the competition between representing information within view and that in the active state of visual WM may be derived from the early visual areas. This is due to the fact that representing visibly present stimuli and memory contents in the active state was highly dependent on the visual cortex^11,12,14,15^. Thus, the visual cortex might work in a similar way to the “competitive content maps” proposed by Franconeri *et al*.^48^. According to their view, the “representational” mechanism underlain by the visual cortex may operate in a “two-dimensional map” architecture in which multiple items competed for the limited cortical real estate^48^, independent of the property of stimuli. Existing evidence suggested that this competition may stem from a competitive inhibition mechanism between representations in the visual cortex. A recent study by Kiyonaga and Egner seems to provide direct evidence for this view. They found that the surrounding suppression occurring in the perceptual scene could also be observed between the visual WM contents and the perceptual input^49^. It’s worth noting that early visual areas have been identified as the neural origin of surrounding suppression^50–52^. Another phenomenon resulting from competitive inhibition in the early visual cortex is visual crowding^53^, which is manifested as the inhibition of perceptual representation on the target stimulus by the adjacent stimuli^54,55^. Interestingly, Tamber-Rosenau and his collaborators demonstrated that visual crowding can also be observed between visual WM contents^56^. These literatures suggested that the limited capacity of the “representational” mechanism proposed by Tsubomi *et al*.^10^ may also be derived from the competitive inhibition in the visual cortex, or from the combined effect of top-down attention control and bottom-up visual cortex competition. Unfortunately, the current data cannot provide direct evidence for this view. We suggest future studies directly examine the relationship between the limited capacity of “representational” mechanism and the competitive inhibition at the visual cortex level.

## Data Availability

The datasets generated during and/or analysed during the current study are available from the corresponding author (lq780614@163.com, Qiang Liu) on reasonable request.

## References

[1] Cowan, N. An embedded process model of working memory. In *Models of working memory: Mechanisms of active maintenance and executive control* 62–101 (1999).

[2] Zhang W, Luck SJ (2008). Discrete fixed-resolution representations in visual working memory. Nature.

[3] Desimone R, Duncan J (1995). Neural Mechanisms of Selective Visual Attention. Annu. Rev. Neurosci..

[4] Scalf PE, Beck DM (2010). Competition in Visual Cortex Impedes Attention to Multiple Items. J. Neurosci..

[5] Franconeri SL, Jonathan SV, Scimeca JM (2010). Tracking multiple objects is limited only by object spacing, not by speed, time, or capacity. Psychol. Sci..

[6] Storm RW, Pylyshyn ZW (1988). Tracking multiple independent targets: Evidence for a parallel tracking mechanism. Spat. Vis..

[7] Yantis S (1992). Multielement visual tracking: Attention and perceptual organization. Cogn. Psychol..

[8] Pylyshyn ZW (2000). Situating vision in the world. Trends Cogn. Sci..

[9] Pylyshyn ZW (2004). Some puzzling findings in multiple object tracking: I. Tracking without keeping track of object identities. Vis. cogn..

[10] Tsubomi H, Fukuda K, Watanabe K, Vogel EK (2013). Neural Limits to Representing Objects Still within View. J. Neurosci..

[11] Moore T, Zirnsak M (2017). Neural Mechanisms of Selective Visual Attention. Annu. Rev. Psychol..

[12] Kastner S (2001). Modulation of sensory suppression: Implications for receptive field sizes in the human visual cortex. J. Neurophysiol..

[13] Harrison SA, Tong F (2009). Decoding reveals the contents of visual working memory in early visual areas. Nature.

[14] Serences JT, Ester EF, Vogel EK, Awh E (2009). Stimulus-specific delay activity in human primary visual cortex. Psychol. Sci..

[15] Scimeca JM, Kiyonaga A, D’Esposito M (2018). Reaffirming the Sensory Recruitment Account of Working Memory. Trends Cogn. Sci..

[16] Emrich SM, Riggall AC, La Rocque JJ, Postle BR (2013). Distributed patterns of activity in sensory cortex reflect the precision of multiple items maintained in visual short-term memory. J. Neurosci..

[17] Ester EF, Anderson DE, Serences JT, Awh E (2013). A Neural Measure of Precision in Visual Working Memory. J. Cogn. Neurosci..

[18] McElree B (1998). Attended and Non-Attended States in Working Memory: Accessing Categorized Structures. J. Mem. Lang..

[19] Oberauer K (2002). Access to Information in Working Memory: Exploring the Focus of Attention. J. Exp. Psychol. Learn. Mem. Cogn..

[20] Larocque, J. J., Lewis-peacock, J. A. & Postle, B. R. Multiple neural states of representation in short-term memory? It’ s a matter of attention. *Front*. *Hum*. *Neurosci*. **8** (2014). 10.3389/fnhum.2014.00005 PMC3899521 24478671

[21] LaRocque JJ, Lewis-Peacock JA, Drysdale AT, Oberauer K, Postle BR (2013). Decoding attended information in short-term memory: an eeg study. J. Cogn. Neurosci..

[22] Lewis-Peacock JA, Drysdale AT, Oberauer K, Postle BR (2012). Neural Evidence for a Distinction between Short-term Memory and the Focus of Attention. J. Cogn. Neurosci..

[23] Zokaei N, Manohar S, Husain M, Feredoes E (2014). Causal Evidence for a Privileged Working Memory State in Early Visual Cortex. J. Neurosci..

[24] Christophel TB, Iamshchinina P, Yan C, Allefeld C, Haynes JD (2018). Cortical specialization for attended versus unattended working memory. Nat. Neurosci..

[25] Stokes MG (2015). ‘Activity-silent’ working memory in prefrontal cortex: A dynamic coding framework. Trends Cogn. Sci..

[26] Mongillo G, Barak O, Tsodyks M (2008). Synaptic theory of working memory. Science (80-.)..

[27] Rose NS (2016). Reactivation of latent working memories with transcranial magnetic stimulation. Science (80-.)..

[28] Luck SJ, Vogel EK (1997). The capacity of visual working memory for features and conjunctions. Nature.

[29] Buschman TJ, Siegel M, Roy JE, Miller EK (2011). Neural substrates of cognitive capacity limitations. Proc. Natl. Acad. Sci..

[30] Vogel EK, Machizawa MG (2004). Neural activity predicts individual differences in visual working memory capacity. Nature.

[31] Luria R, Balaban H, Awh E, Vogel EK (2016). The contralateral delay activity as a neural measure of visual working memory. Neurosci. Biobehav. Rev..

[32] Adam KCS, Robison MK, Vogel EK (2018). Contralateral delay activity tracks fluctuations in working memory performance. J. Cogn. Neurosci..

[33] Cavanagh JF, Frank MJ (2014). Frontal theta as a mechanism for cognitive control. Trends Cogn. Sci..

[34] Adam KCS, Mance I, Fukuda K, Vogel EK (2015). The contribution of attentional lapses to individual differences in visual working memory. J. Cogn. Neurosci..

[35] Kronland-Martinet, R., Morlet, J. & Grossmann, A. Analysis of sound patterns through wavelet transforms. *Int. J. Pattern Recognit. Artif. Intell.* **1,** 97–126 (1987)

[36] Maris E, Oostenveld R (2007). Nonparametric statistical testing of EEG- and MEG-data. J. Neurosci. Methods.

[37] Cohen, J. *Statistical power analysis for the behavioral sciences*. (1977).

[38] Cumming G, Fidler F (2009). Confidence intervals better answers to better questions. J. Psychol..

[39] Gilchrist, I. D. & Harvey, M. Refixation frequency and memory mechanisms in visual search. *Curr. Biol.* **10,** 1209–1212 (2000). 10.1016/s0960-9822(00)00729-6 11050390

[40] Peterson, M. S., Kramer, A. F., Wang, R. F., Irwin, D. E. & McCarley, J. S. Visual search has memory. *Psychol. Sci*. **12,** 287–292 (2001). 10.1111/1467-9280.00353 11476094

[41] Larocque, J. J., Riggall, A. C., Emrich, S. M. & Postle, B. R. Within-Category Decoding of Information in Different Attentional States in Short-Term Memory. *Cereb. Cortex* **27**, 4881–4890 (2017). 10.1093/cercor/bhw283 PMC6059111 27702811

[42] Woodman GF, Vogel EK (2005). Fractionating working memory: consolidation and maintenance are independent processes. Psychol. Sci..

[43] Shaffer W, Shiffrin RM (1972). Rehearsal and storage of visual information. J. Exp. Psychol..

[44] Quentin, R. *et al*. Differential brain mechanisms of selection and maintenance of information during working memory Differential brain mechanisms of selection and maintenance of information during working memory Abstract Working memory is our ability to select and temporaril. *J*. *Neurosci*. (2019). 10.1523/JNEUROSCI.2764-18.2019 PMC6510345 30833510

[45] Gunseli, E. *et al*. Unattended but actively stored: EEG dynamics reveal a dissociation between selective attention and storage in working memory. *bioRxiv* 320952), 10.1101/320952 (2018).

[46] Noudoost, B., Chang, M. H., Steinmetz, N. A. & Moore, T. Top-down control of visual attention. *Current Opinion in Neurobiology*, 10.1016/j.conb.2010.02.003 (2010). 10.1016/j.conb.2010.02.003 PMC2901796 20303256

[47] Zavala, B. *et al*. Subthalamic nucleus local field potential activity during the eriksen flanker task reveals a novel role for theta phase during conflict monitoring. *J*. *Neurosci*., 10.1523/JNEUROSCI.1036-13.2013 (2013). 10.1523/JNEUROSCI.1036-13.2013 PMC3771028 24027276

[48] Franconeri SL, Alvarez GA, Cavanagh P (2013). Flexible cognitive resources: Competitive content maps for attention and memory. Trends Cogn. Sci..

[49] Kiyonaga A, Egner T (2016). Center-Surround Inhibition in Working Memory. Curr. Biol..

[50] Müller NG, Kleinschmidt A (2004). The attentional ‘spotlight’s’ penumbra: Center-surround modulation in striate cortex. Neuroreport.

[51] Boehler CN, Tsotsos JK, Schoenfeld MA, Heinze HJ, Hopf JM (2009). The center-surround profile of the focus of attention arises from recurrent processing in visual cortex. Cereb. Cortex.

[52] Boehler CN, Tsotsos JK, Schoenfeld MA, Heinze HJ, Hopf JM (2011). Neural mechanisms of surround attenuation and distractor competition in visual search. J. Neurosci..

[53] Millin R, Arman AC, Chung STL, Tjan BS (2014). Visual crowding in V1. Cereb. Cortex.

[54] Chen J (2014). Attention-dependent early cortical suppression contributes to crowding. J. Neurosci..

[55] Bouma H (1970). Interaction effects in parafoveal letter recognition. Nature.

[56] Tamber-rosenau BJ, Fintzi AR, Marois R (2015). Crowding in Visual Working Memory Reveals Its Spatial Resolution and the Nature of Its Representations. Psychol. Sci..

